# Recent Advances in Biochar Polymer Composites

**DOI:** 10.3390/polym14122506

**Published:** 2022-06-20

**Authors:** Mattia Bartoli, Rossella Arrigo, Giulio Malucelli, Alberto Tagliaferro, Donatella Duraccio

**Affiliations:** 1 Center for Sustainable Future Technologies, Italian Institute of Technology, Via Livorno 60, 10144 Turin, Italy; mattia.bartoli@polito.it; 2 Department of Applied Science and Technology, Politecnico di Torino, Viale Teresa Michel 5, 15121 Alessandria, Italy; giulio.malucelli@polito.it; 3 Department of Applied Science and Technology, Politecnico di Torino, Corso Duca degli Abruzzi 24, 10123 Torino, Italy; alberto.tagliaferro@polito.it; 4 Institute of Sciences and Technologies for Sustainable Energy and Mobility, National Council of Research, Strada delle Cacce 73, 10135 Torino, Italy; donatella.duraccio@stems.cnr.it

**Keywords:** biochar, zero-waste approach, circular economy, polymer-based composites, mechanical properties, electrical properties

## Abstract

“Biochar” (BC) is the solid residue recovered from the thermal cracking of biomasses in an oxygen-poor atmosphere. Recently, BC has been increasingly explored as a sustainable, inexpensive, and viable alternative to traditional carbonaceous fillers for the development of polymer-based composites. In fact, BC exhibits high thermal stability, high surface area, and electrical conductivity; moreover, its main properties can be properly tuned by controlling the conditions of the production process. Due to its intriguing characteristics, BC is currently in competition with high-performing fillers in the formulation of multi-functional polymer-based composites, inducing both high mechanical and electrical properties. Moreover, BC can be derived from a huge variety of biomass sources, including post-consumer agricultural wastes, hence providing an interesting opportunity toward a “zero waste” circular bioeconomy. This work aims at providing a comprehensive overview of the main achievements obtained by combining BC with several thermoplastic and thermosetting matrices. In particular, the effect of the introduction of BC on the overall performance of different polymer matrices will be critically reviewed, highlighting the influence of differently synthesized BC on the final performance and behavior of the resulting composites. Lastly, a comparative perspective on BC with other carbonaceous fillers will be also provided.

## 1. Introduction

Environmental safety and the progressive depletion of fossil fuel-based sources are currently a great concern for both academic and industrial research. As a result, there is an increasing interest in sustainable manufacturing [1,2,3]. The investigation of new eco-sustainable and bio-based composites has gained great attention, especially concerning eco-friendly systems derived from waste and renewable resources [4]. Accordingly, a promising alternative to conventional carbonaceous fillers is biochar (BC), a carbonaceous and renewable material produced by the thermo-chemical conversion of biomasses in an oxygen-limited environment [5,6,7]. Unlike other carbon-based materials, BC is derived from sustainable biomass resources and possesses high thermal stability and hardness, high surface area, good chemical stability, and electrical conductivity [8,9,10,11]. Up to now, BC has been widely investigated for environmental remediation [12,13,14], as catalyst support [15], and for energy storage applications [16]. Nevertheless, BC-based composites still require optimization to reach performance comparable to traditional carbon-based fillers such as graphene and carbon nanotubes. These materials can be used to reach great composites performance, but they are costly. In 2020, the price of single layer graphene was higher than USD 230/cm^2^, while for graphene oxide the price was USD 140/kg. Conversely, carbon black was sold for around USD 1.2/kg [8]. Compared to a cheap carbon filler such as carbon black, BC has a lower cost and is derived from biomass.

In this context, investigations dealing with the formulation of BC-containing polymeric systems based either on thermoplastic or thermosetting matrices have been increasing exponentially in recent years. Considering its intriguing characteristics, along with the possibility to tailor its structure and functionalization, BC represents an attractive alternative to traditional carbonaceous fillers for improving the mechanical, electrical, and physical properties of polymer-based composites [4,17,18]. 

Nevertheless, the relation between the properties of BC and those of its composites is hard to establish, mainly due to the great BC variability [19]. The spread of the use of BC for the production of polymeric composites brings about the need of a reference point for both the specialists and the newcomers in the field. The present review aims at providing a comprehensive overview of BC-containing composites based on both thermoplastic and thermosetting polymers, highlighting the main achievements reached in the last decade. In particular, we discuss the relationship between BC properties (i.e., particle size, functionalization, and graphitization degree) and the properties of resulting composites. Finally, a comparative perspective on BC with other carbonaceous fillers will also be provided.

## 2. A Brief Overview of BC Production and Properties

BC is produced through thermochemical cracking of biomasses following three main routes named hydrothermal liquefaction, pyrolysis, and gasification. 

Hydrothermal liquefaction is a thermochemical conversion operating in a temperature range up to 350 °C, in a water medium, and under moderate pressure. This procedure promotes advanced depolymerization of biomass giving rise to highly functionalized BC named hydrochar [20]. 

Proper pyrolytic processes take place at temperatures above 400 °C in an oxygen-limited [21] or inert atmosphere [7]. By using the pyrolytic approach, it is possible to achieve a fast and advanced cracking process of each biomass component (lignin, cellulose, and hemicellulose) with the simultaneous production of BC, bio-oils, and non-condensable gases [22] with a wide variation in fraction yields based on heating technologies [21,23,24,25,26] and plant design [7,27].

Gasification is the other route for BC production that is run in an oxidant atmosphere by using air [28], oxygen, or even steam [29] with temperatures higher than 800 °C. The combination of high temperature and oxidant atmosphere induces the conversion of biomass into a gas mixture mainly composed of hydrogen, methane, carbon dioxide, carbon monoxide, and steam. The solid output of gasification is a BC with a very high ash content and low carbon percentage. In a common pyrolytic process, biomass undergoes proper carbonization at temperatures ranging from 300 °C to 400 °C with cracking of its components through complex reaction routes and forming. In this stage, BC is massively tailored with oxygen-based functionalities (i.e., hydroxyl, carbonyl, and carboxylic residues) and displays a highly defective carbon structure. By increasing the temperature from 600 °C to 800 °C, the aromatic structures further condense, forming proper graphite-like domains, still highly disordered but with less residual groups. These materials are commonly classified as hard carbon due to their high mechanical hardness [30]. Further temperature increments lead to a progressive enlargement and ordering process of graphitic domains through turbostatical rearrangement [31] that ends at about 3000 °C when the maximum graphitization degree is reached [32]. As widely discussed by Weber et al. [33], the properties of BC (i.e., surface area, porosity, grindability, etc.) originated from a complex combination of interactions due to the morphology and chemical composition of the feedstock and can be tailored by post-treatments such as surface tailoring or activation [34].

The technology chosen for BC production plays a crucial role in determining the final properties of the filler, and it is related to a complex combination of economic and strategic features. All properties of BC are simultaneously affected by all the selected process parameters such as production temperature, reactor design, and feedstock used [35], and it is hard to establish systematic and general rules for their simultaneous optimization. Nevertheless, the quality of BC can be ensured for large scale-production over time [36]. 

As far as the influence of feedstock is concerned, it has been demonstrated that wood-derived BC exhibits a highly volatile content compared to materials obtained from non-woody sources. Furthermore, depending on the type of feedstock, a variation in the quality and amount of heteroatoms and metal elements incorporated into BC has been observed [37]. 

In general, an increase in pyrolysis temperature, apart from the already discussed structural modifications, causes a decrease in the content of functional groups, thus affecting the affinity of the obtained BC toward polar moieties. In particular, a concurrent decrease of the O/C and N/C ratios is usually observed in BC pyrolyzed at high temperatures due to the occurrence of temperature-induced dehydration and decarboxylation processes [38,39]. Furthermore, pyrolysis processes performed at high temperatures induce an improvement in the solvent absorption capability of BC, because of the formation of nonporous structures [40]. 

Finally, it has been shown that the reactors employed for BC production have a marginal effect on the elemental carbon content, surface functionalities, and thermal degradation of BC. Interestingly, Das et al. reported a significant effect of the pyrolysis reactor on the fire resistance of the resulting BC. In particular, they showed that BC obtained in a hydrothermal reactor exhibits high fire resistance due to the presence of tarry volatiles, which are able to seal water molecules within the BC pores, thus hindering the material combustion [41].

## 3. BC-Based Composites: Properties and Applications

### 3.1. Polyolefins-Based Composites

#### 3.1.1. Polypropylene (PP)-Based Composites

The first pioneering works related to the utilization of BC derived from pyrolysis processes of organic wastes were conducted by Das et al. [42], who exploited BC in combination with wood to enhance the final properties of wood–plastic composites (WPCs). In fact, WPCs usually present some disadvantages such as thickness swelling, thermal instability, and low interfacial bonding, which can be profitably overcome through the addition of BC particles [17]. In particular, BC filler produced from landfill pine wood at two different pyrolysis temperatures, namely 400 and 450 °C, was added to a PP matrix through melt extrusion at different loadings (ranging from 6 to 30 wt.%), together with 30 wt.% of wood. The mechanical characterization of the composites revealed that the material containing 24 wt.% of BC exhibits similar tensile strength and modulus but higher flexural properties compared to conventional wood/PP composites. These findings were attributed to the improvement of the interfacial adhesion between wood and PP because of the BC incorporation. However, a severe reduction of the material ductility was observed for BC loadings beyond 15 wt.%. 

**Figure 1:**
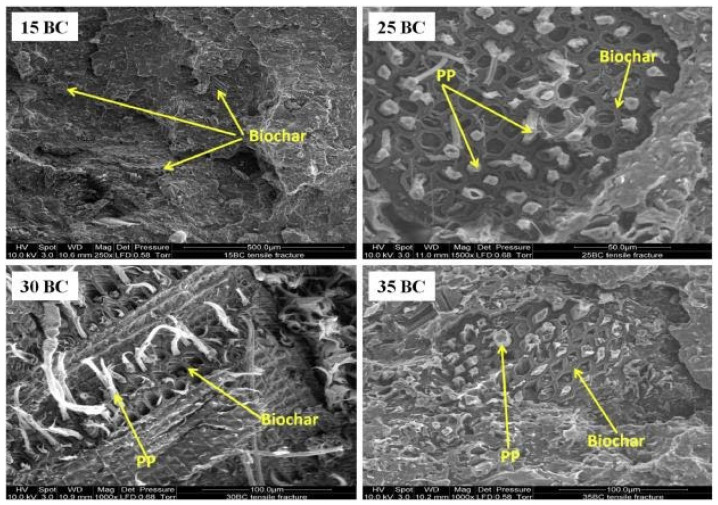
SEM micrographs of PP/BC composites containing different amounts of filler [44]. Reproduced with permission from Elsevier Science Ltd. 2016, Elsevier Science, Ltd.

Aiming at assessing the possible influence of the waste feedstock on the main characteristics (such as surface area, carbon and ash content, and pore volume among a few to mention) of the obtained BC particles and, consequently, on the final properties of the resulting PP-based composites, the same research group exploited six types of BC coming from different waste sources to formulate WPCs containing 30 wt.% of wood and 24 wt.% of BC [43]. From a general point of view, the incorporation of BC enhanced PP tensile and flexural moduli and strength compared to WPC counterparts. Based on the obtained results, the authors concluded that mineral/carbon content and particle surface area are the main factors affecting the mechanical properties of the resulting composites. In fact, the composites containing BCs with higher carbon content and larger surface area exhibited higher values of tensile strength and moduli. Furthermore, the characterization of the fire properties of the composites indicated that the systems containing BC with high CaCO_3_ loadings exhibited a lower heat release rate compared to other composites due to the beneficial effect of the inorganic content in hindering the diffusion path of the oxygen in the composite during forced-combustion tests. This result revealed the opportunity to impart fire retardance to polymer-based composites with the introduction of BC particles; this finding was further verified for PP-based composites containing up to 30 wt.% of BC obtained from landfill pine wood waste by pyrolysis at 500 °C and a subsequent high temperature (900 °C) activation process. For these composites, the peak of the heat release rate and the smoke production rate were lowered by increasing the BC content [44]. In addition, in this case, the improved fire performance of the composites were attributed to the formation of a compact carbonaceous layer, preventing the oxygen transfer toward the PP matrix. Furthermore, the authors demonstrated that the introduction of increasing loadings of BC caused a monotonic increase of the tensile and flexural moduli due to the peculiar morphology of the composites. In detail, as shown by the SEM micrographs presented in Figure 1, the molten PP chains were able to infiltrate the porous structure of the BC particles during the processing, resulting in the formation of an extended network of mechanical interlocking between the polymer matrix and the embedded filler, hence increasing flexural and tensile moduli. 

Further studies have documented that a fundamental role in obtaining composite materials with superior mechanical properties was played by the physical bonding achieved through the infiltration of the polymer chains into the BC pores, eliminating the need to use coupling agents for the enhancement of the interfacial adhesion between BC particles and the polymer matrix [45,46]. 

The beneficial effect of the BC incorporation on the fire behavior of PP was also explored in the presence of other conventional flame retardant compounds, such as ammonium polyphosphate and magnesium hydroxide [47,48]. In particular, composites containing different loadings of BC particles obtained from landfill pine wood, wood fibers, and 20 wt.% of flame retardants exhibited significantly enhanced fire retardant performance with respect to the unfilled matrix. However, the simultaneous presence of BC and the selected flame retardants induced some detrimental effects on the mechanical performance of the composites, since flame retardant particles were trapped within BC pores, thus hindering the flow of the polymer chains and reducing the effectiveness of the mechanical interlocking between PP macromolecules and BC particles.

An interesting study performed by Das et al. [49] demonstrated the possibility to predict the mechanical performance of PP-based composites containing BC produced from the pyrolysis of waste pine wood by means of a nanoindentation study on both the individual particles and the polymer. In detail, the measured nanoindentation values for hardness and moduli were exploited together with theoretical models to compare the predicted and experimental results, showing a good agreement between experimental values and theoretical predictions. It evidenced the fundamental role of the polymer infiltration phenomenon, which is responsible for higher hardness values for the composites compared to bare BC particles. 

In a recent study, Paleri et al. [50] showed that the proper selection of the BC pyrolysis conditions, especially the treatment temperature, significantly affects the mechanical performance of the resulting composites. More specifically, BC particles derived from distillers’ dried grains with solubles, i.e., a co-product from the corn ethanol industry, were obtained through torrefaction and pyrolysis at different temperatures and exploited as fillers in a PP matrix. Preliminary analyses performed on the BC particles showed a decrease in the functional groups content and an increase in the ash content by increasing the treatment temperature. The mechanical characterization of the composites revealed that the best performance in terms of stiffness–toughness balance were achieved for the system containing BC pyrolyzed at 700 °C. In particular, the introduction of this type of BC caused an improvement in elastic modulus (+40%), flexural modulus (+55%), and impact strength (+27%) compared to the unfilled PP. Furthermore, the rheological characterization of the formulated systems demonstrated the obtainment of higher complex viscosity and storage modulus values for the system containing BC pyrolyzed at 700 °C with respect to the others, indicating an improved compatibility with PP macromolecules. The best performance achieved by BC particles pyrolyzed at 700 °C were ascribed to their low polarity compared to those obtained at lower temperatures (i.e., at 500 or 600 °C) and to their lower ash content and particle size with respect to the BC particles obtained at 1000 °C. However, Ayadi et al. [51] demonstrated that the decrease of the number of functional groups resulting from pyrolysis performed at high temperatures severely limited the obtainment of superior mechanical properties. More specifically, they investigated PP-based composites containing BC particles derived from wood at different pyro-gasification temperatures and a coupling agent. Pyrolysis processes carried out at temperatures beyond 400 °C induced the presence of progressively lower amounts of functional groups, hence preventing the achievement of strong interfacial interactions between the coupling agent and the BC particles. On the other hand, the modification of BC chemical structure induced by the treatment at high temperatures promoted a remarkable increase of the dimensional stability of the composites and an improvement of their hydrophobicity. 

**Figure 2:**
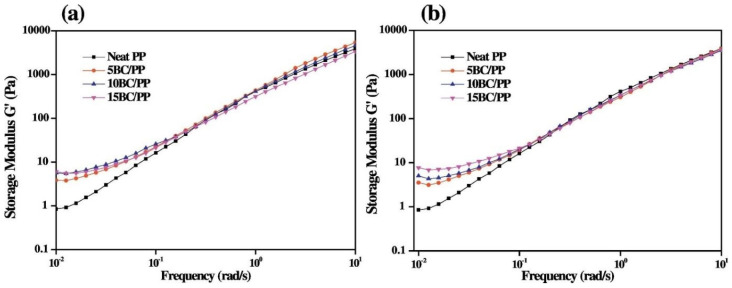
Storage modulus as a function of frequency for PP-based composites containing BC particles obtained at: (**a**) 700 °C and (**b**) 900 °C [52]. Reproduced with permission from Elsevier Science Ltd. 2018, Elsevier Science Ltd.

Despite the role of the rheological characterization in providing an indirect evaluation of the extent of filler distribution and of possible polymer/filler interactions established at the interface, few works discuss the effect of BC introduction on the PP rheological response [50,52,53,54,55,56]. In particular, Poulose et al. [52,57] investigated the rheological properties of PP-based composites containing different loadings of BC particles derived from date palm waste through a pyrolysis process performed at two temperatures, namely 700 and 900 °C. The obtained results (Figure 2) indicated a different rheological behavior for the composites compared to unfilled PP, especially in the low frequency region; more specifically, the flattening of the moduli curves highlighted the formation of an extended filler–filler network within the matrix, causing the restriction of the motion of PP macromolecules. However, the low increment of the storage modulus values for the composites compared to those of the unfilled matrix indicated a poor degree of interaction between the embedded particles and PP macromolecules. Furthermore, the analysis of the tanδ curves as a function of frequency suggested that in the formulated composites the rheological percolation threshold was not reached for all investigated BC loadings, likely due to the weak PP–BC interactions. 

Furthermore, it was demonstrated that the introduction of BC within a PP matrix remarkably affects the crystallization behavior of the resulting composites. In fact, usually PP–BC composites show a higher crystallinity degree compared to the unfilled matrix due to the nucleating action exerted by BC particles [58]. In this context, Elnour et al. [59] documented a progressive increase of the crystallization temperature with increasing BC loading, due to the rising number of nucleation sites available for the crystallization; a correlation between the composite crystallization temperature and the pyrolysis temperature of BC was observed. 

**Figure 3:**
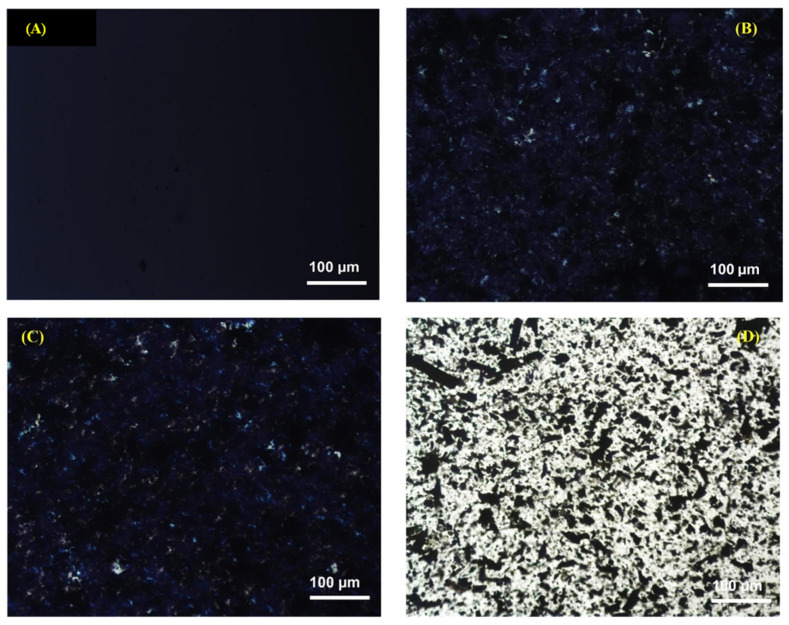
POM images of PP-based composite containing 15 wt.% of BC obtained at 500 °C; images taken at: (**A**) 0 min; (**B**) 2 min, (**C**) 5 min; and (**D**) 5 min without cross polarizer [57]. Reprinted under CC BY 4.0 license.

Analogously, Alghyamah et al. [57] evaluated the crystallization behavior of PP–BC composites containing different loadings (namely, 5, 10, 15, and 20 wt.%) of carbonaceous particles prepared from waste biomass, with pyrolysis temperatures ranging from 300 to 700 °C. The obtained results revealed that the introduction of BC particles enhanced the overall crystallization process of the composites. Furthermore, the assessment of the crystallization kinetics documented that the overall crystallization and nucleation rates were enhanced in the presence of BC particles. The analysis of the data through the Avrami model [60] allowed discriminating two different BC characteristics, dependent on the pyrolysis temperature, strongly affecting the PP crystal formation, namely the porous structure and the surface area. In detail, the BC prepared at higher temperatures promoted the growth of PP crystals having a two-dimensional disk-like shape, while the BC obtained at lower temperatures induced the formation of rod-like structures. These differences were associated with the highly infiltrated morphology obtained in the composites containing BC particles prepared at higher temperatures. Additionally, the evaluation of the spherulitic growth (Figure 3) carried out at a crystallization temperature of 120 °C pointed out the formation of a significantly high number of crystalline structures having smaller dimensions compared to those observed in unfilled PP. This phenomenon was associated with the fast heterogeneous nucleation occurring in the presence of BC particles, resulting in the formation of small-sized PP spherulites with imperfect morphologies.

#### 3.1.2. Polyethylene (PE)-Based Composites

Among PEs, U\ultra-H\high M\molecular W\weight P\9olyethylene (UHMWPE) was often selected as the polymer matrix for preparing composites containing BC, aiming at obtaining conductive materials suitable for applications as antistatic materials and sensors [61]. The use of UHMWPE as the polymer matrix for conductive polymer composites is well documented in the literature and is justified by the segregated microstructure usually obtained in UHMWPE-based composites, which can effectively reduce the interfacial electrical resistance between the filler and the polymer matrix due to the development of a continuous conductive path leading to an improvement of the electrical conductivity of the material [62]. From a general point of view, conductive polymer-based materials are often preferred to metallic conductors due to their wide range of electrical conductivity and, in particular, to their lower cost. Although various carbon-based fillers such as graphene [63,64] and carbon nanotubes [65,66] have been used to formulate UHMWPE-based conductive polymer composites, these nanoparticles are expensive and non-sustainable; therefore, there is a rising interest in exploiting BC as a cheap and bio-sustainable alternative to these materials. In this context, composites systems based on polyethylene and containing different types of BC have been designed and formulated, aiming at assessing the effect of the BC introduction on the electrical and also the mechanical properties of the resulting composites [67]. 

**Figure 4:**
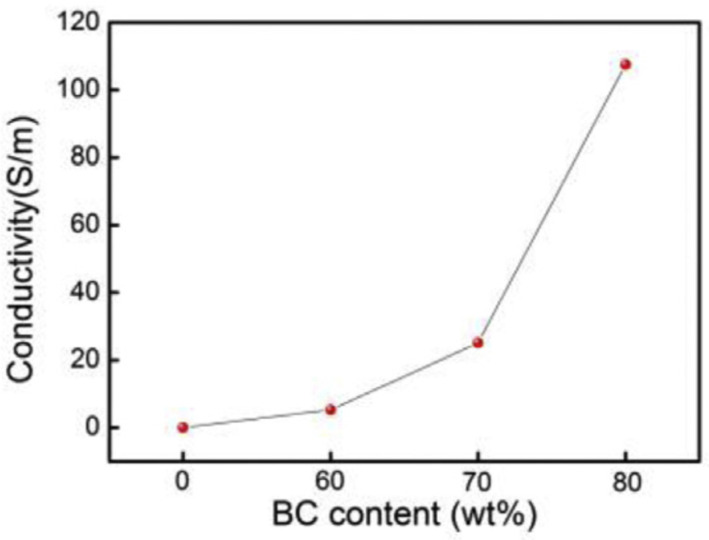
Electrical conductivity as a function of the content of commercial BC particles derived from Bamboo charcoal for UHMWPE/LLDPE composites [68]. Reproduced with permission from Elsevier Science Ltd. 2018, Elsevier Science Ltd.

Generally, the incorporation of BC into UHMWPE allows obtaining composite materials with high mechanical properties and high electrical conductivity [61,68]. In this context, Li et al. [68] prepared highly filled (up to 80 wt.%) composites using commercial BC particles derived from Bamboo charcoal, through melt extrusion. Due to its high molecular weight and consequent very high viscosity, UHMWPE is not processable through extrusion; for this reason, a blend of UHMWPE and linear low-density polyethylene (LLDPE) was used as the matrix. The evaluation of the electrical properties of the obtained composites demonstrated that the BC conductive pathways were well established for filler loadings beyond 60 wt.%, and the electrical conductivity of the materials progressively increased as a function of the BC loading (see Figure 4). In particular, the composite containing 80 wt.% of BC exhibited a conductivity of 107.6 S/m. This excellent electrical performance was attributed to a mechanism involving the electron transfer between BC particles by direct physical contact or through a special tunneling phenomenon. Furthermore, it was demonstrated that high BC loadings are effective in providing large amounts of free electrons to attenuate the electromagnetic radiation; the composite containing 80 wt.% of BC showed a very high EMI shielding effectiveness, indicating that BC has a great potential as a filler for conductive polymer-based composites for a variety of engineering and electrical applications. 

Flexible UHMWPE/BC composites with tunable conductivity and good mechanical properties prepared using extrusion and hot-compression methods were obtained by Li et al. [69] by exploiting three different types of BC particles (derived from commercial samples of pine, apple, and bamboo charcoal) obtained through a pyrolysis process within 500 and 1100 °C. The assessment of the material morphology indicated the achievement of a uniform dispersion of all kinds of BC within UHMWPE and the establishment of strong polymer/filler interfacial interactions. It was demonstrated that an increase in the pyrolysis temperature promotes a modification of the electrical behavior of the BC, switching from insulating to conductive material. Finally, for the composites containing 70 wt.% of BC obtained at the highest carbonization temperature, high values of electrical conductivity were recorded (namely, 3.0 × 10^−1^, 3.7 × 10^−1^, and 3.9 × 10^−1^ S/cm for the composites containing BC from pine, apple, and bamboo charcoal, respectively), proving the suitability of these materials for many electrical applications. In addition, in this case, the conductive mechanism was attributed to the formation of BC conductive networks throughout the matrix, promoting electron transfer between contiguous particles. 

**Figure 5:**
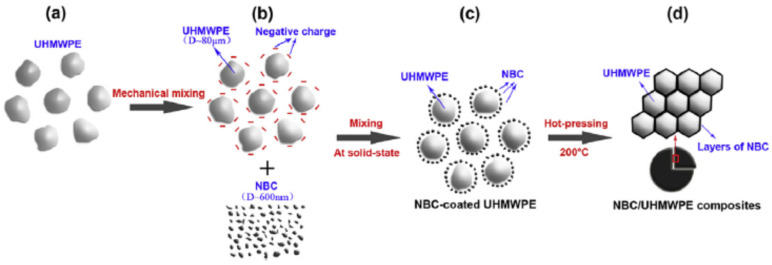
(**a**–**d**) Schematic representation of the preparation method for composites with segregated morphology [70]. Reproduced with permission from Elsevier Science Ltd. 2016, Elsevier Science Ltd.

Li et al. [70] demonstrated that low values of the electrical percolation threshold can be obtained in UHMWPE-based composites showing a fully-developed segregated morphology. More specifically, exploiting a combination of high-speed mechanical mixing and hot compaction (see the schematic representation depicted in Figure 5), the BC particles are distributed only at the interface between contiguous polymer granules; this way, a segregated conductive network is achieved with lower BC content compared to similar composites showing a uniform filler distribution, leading to the obtainment of a low electrical percolation threshold. 

A typical percolation behavior was observed from the evaluation of the electrical conductivity of the segregated UHMWPE/BC composites as a function of the BC content, with an increase of nearly 10 orders of magnitude between 0.8 and 2.3 vol.% of BC; for these systems, the value of the percolation concentration was found to be 2.0 vol.%. The formulated composites exhibited increased thermal stability and tensile strength compared to the unfilled matrix, confirming the applicability of the proposed processing route for the formulation of BC-based materials suitable for industrial applications. 

Quite recently, UHMWPE-BC composites containing high loadings of BC obtained from commercial bamboo charcoal particles pyrolyzed at 800 and 1000 °C were proposed for orthopedic applications [71]. Composite materials containing BC pyrolyzed at 1000 °C showed improved hardness and tensile modulus values. Conversely, the introduction of BC obtained at lower pyrolysis temperature enhanced tensile strength and wettability due to a greater polymer/filler affinity, as well as a lower friction coefficient and higher biocompatibility compared to unfilled UHMWPE, highlighting the potential of this material to be exploited for orthopedic applications. 

High-density polyethylene (HDPE) was widely utilized as the polymer matrix for BC-containing composites. In particular, HDPE–BC composites were specifically designed for obtaining sustainable materials showing adequate mechanical properties for applications in packaging and the automotive sector [72,73]. In this context, Zhang et al. [74] used BC derived from agricultural wastes as reinforcing filler for melt-extruded composites, aiming at achieving a material with superior mechanical, thermal, and flame retardant properties. It was documented that increasing BC loadings promoted enhanced tensile properties, creep resistance, and anti-stress relaxation ability compared to the unfilled matrix. Furthermore, a progressive improvement of the thermal and flame retardant properties of the composites was documented as a function of the BC content due to its intrinsic higher stability, notwithstanding a slight decrease of the water resistance of the composites with respect to unfilled HDPE. 

Zhang et al. [75] showed that the pyrolysis conditions undergone by BC particles have a dramatic influence on the final properties of HDPE–BC composites. More specifically, they demonstrated that an increase of the treatment temperature from 200 to 700 °C caused an increase in specific surface area and pore volume along with a decrement of the content of polar groups and, hence, of the polarity of the BC particles. As a result, composites containing BC particles obtained at higher temperatures exhibited improved flexural strength due to the higher amount of HDPE macromolecules infiltrated into the porous structure of the filler, allowing the development of an interlocking structure, which made the stress transfer mechanism more efficient. The absence of polar functional groups onto the BC surface led to an improvement of the BC/HDPE compatibility, further inducing the achievement of superior mechanical properties. 

**Figure 6:**
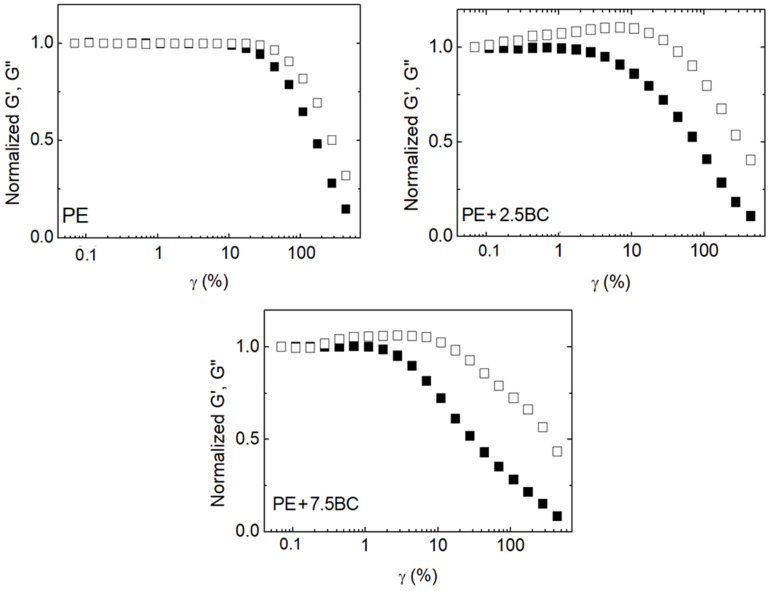
Normalized dynamic moduli (full symbols for G’ and empty symbols for G”) for neat PE and composites with 2.5 and 7.5 wt.% of BC [76]. Reprinted under CC BY 4.0 license.

The strong interactions established in HDPE–BC composites were further evaluated by Arrigo et al. [76] through rheological analyses. In detail, BC particles obtained from waste coffee grounds through pyrolysis at 700 °C were incorporated into HDPE through melt compounding. The analysis of the rheological response of the obtained materials documented a slowing down of the relaxation dynamics of the polymer macromolecules due to the confinement of the polymer chains onto the filler surface and/or within the BC porous structure. Furthermore, a weak strain overshoot behavior in the non-linear regime was observed for the composites containing BC loadings exceeding 2.5 wt.% (Figure 6), indicative of weak structural complexes that oppose the imposed strain. This behavior suggested the establishment of polymer/BC interactions that hinder the motion of the PE macromolecular chains, retarding their complete relaxation.

### 3.2. Polyamide-Based Composites

BC has also been exploited as a reinforcing filler for polyamides. As for polyolefins, BC acts as a rigid filler resulting, in general, in an increased strength and elastic modulus, but remarkably decreasing both ductility and toughness of the composite systems. 

BC-containing composites based on PA6 and the more expensive PA6,10 were the most reported in the literature so far. Ogunsona and co-workers investigated PA6-based composites by using pyrolyzed miscanthus fibers [77], focusing on the effect of BC pyrolysis conditions on the properties of the composites. In detail, miscanthus fibers pyrolyzed at 500 °C and 900 °C at loadings ranging from 6 to 20 wt.% were employed as the matrix reinforcement. The composites containing low temperature (i.e., 500 °C) pyrolyzed BC showed increased tensile and flexural strength values by 19.5% and 31%, respectively, compared to the composites containing high temperature (i.e., 900 °C) pyrolyzed BC. These differences were attributed to the different interfacial adhesion between BC and PA. Conversely, the composites containing BC treated at 900 °C showed lower compatibility due to the limited functional groups present on its surface and available for interacting with the matrix. 

Using a similar approach, Watt et al. [78] analyzed the influence of corn cob BC pyrolyzed at three different temperatures (namely, 350, 500, and 900 °C) on the properties of a commercial PA 4,10. This polyamide is mainly produced by materials derived from natural sources, showing a bio-content of about 76%. The BC employed at 10 and 20 wt.% loading was also ball-milled and sieved to sub-300 μm size. SEM analyses of the composites containing corn cobs pyrolyzed at 350 °C documented the occurrence of a particle encapsulation at the polymer interface resulting in an overall improvement of rheological properties, while the mechanical properties remained relatively constant compared to the other composites. Furthermore, it was shown that the introduction of 20 wt.% of low temperature pyrolyzed BC was optimal, leading to improvement in tensile modulus by 6% while maintaining a low density. Conversely, higher pyrolysis temperatures led to a higher degree of carbonization because of the degradation of functional groups with an overall more disordered structure. This latter, generated by gas evolution, showed a very high density and was responsible for up to a 12% improvement in heat deflection temperature (HDT) when 20 wt.% of BC was incorporated.

In a further research effort, Ogunsona and co-workers [79] studied the influence of the size of BC particles on the properties of PA6,10 composites containing 20 wt.% of filler. Crushed, milled, and fractionated milled biochar particles with size ranges of <63, 213–250, and 426–500 μm, respectively, were used. It was found that as the particle size was reduced, the composites showed increased HDT. This finding was interpreted considering that by reducing BC size, the interparticle distance was also reduced, limiting the freedom or radius of gyration of the polymer macromolecules, especially in the amorphous phase. Therefore, higher temperatures were required to activate flow within the composite, leading to a higher HDT. A similar behavior was found for the impact strength whose value increased by 200% for the composites containing BC particles with a size below 63 mm when compared to those with sizes not exceeding 500 μm. Finally, the addition of big-size BC decreased the impact strength of PA 6, 10 by 70%. More specifically, the impact strength in the composites always remained below that of unfilled PA due to the reduction in ductility and the hindrance in plastic deformation by restricting the dynamics of polymer chains. Interestingly, both flexural strength and modulus were always greater than those of the unfilled matrix but were not correlated with the BC particle size.

A few studies reported the effect of a very high concentration of BC on the thermo-mechanical properties of polyamide 6 [9]. Zhy et al. [80] used commercial bamboo BC pyrolyzed at 1100 °C as filler in PA6. Samples were prepared by melt blending and injection molding, and the amount of BC ranged from 10 to 60 wt.%. It was demonstrated that when the amount of BC was 30 to 40 wt.%, the PA6–BC composites exhibited acceptable properties in terms of strength, toughness, and processing fluidity. 

Ogunsona and co-workers [9] used biochar obtained by miscanthus fibers as a reinforcement in polyamide 6 at loadings up to 40 wt.%. The increase of BC amount was probed to enhance the tensile modulus and HDT values of the composites. At 40 wt.% filler loading, the flexural and tensile strengths increased by 47 and 19.6%, respectively, compared to unfilled polyamide 6. This result was attributed to the good adhesion between BC and polyamide 6 as revealed by scanning electron micrographs of the impact fractured surfaces of the composites. The impact strengths of all the composites remained comparable with that of unfilled polymer. However, when the BC loading was 20 wt.%, the impact strength increased by 43.7% with respect to that of unfilled polyamide 6.

**Figure 7:**
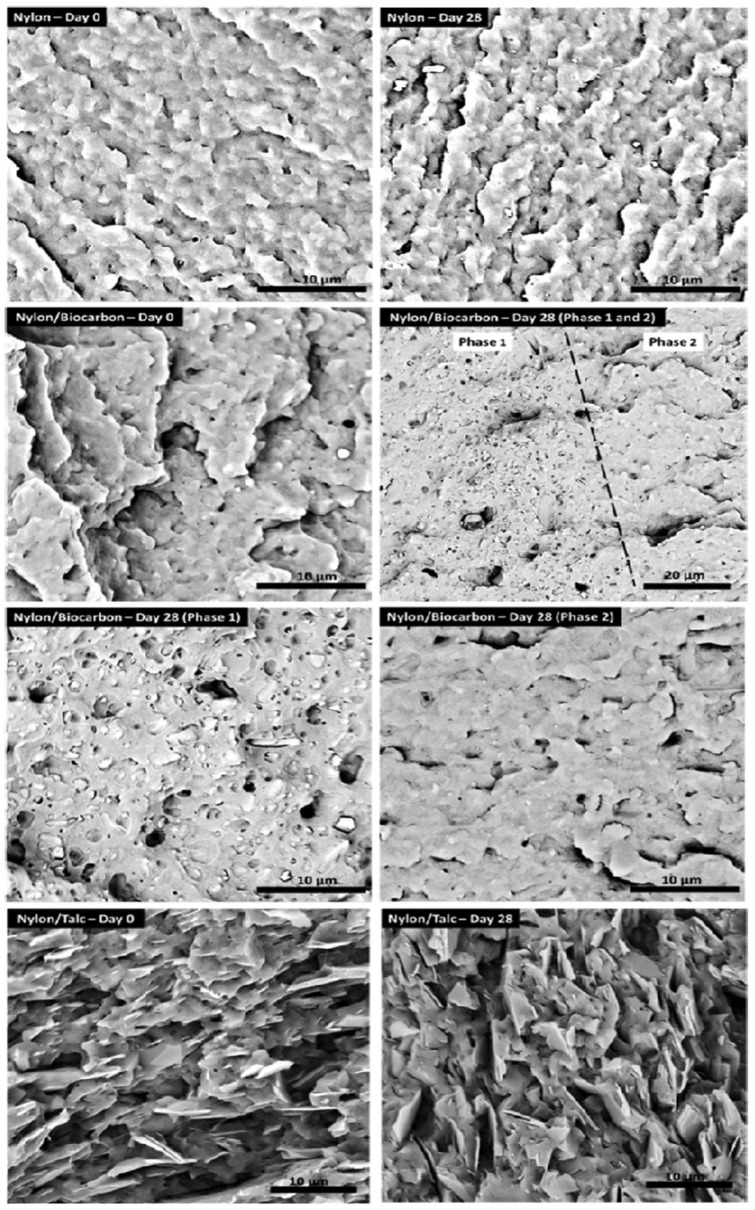
SEM micrographs of the impact fractured surfaces of PA, PA6–BC, and PA6–talc before and after conditioning for 28 days [81]. Reproduced with permission from Elsevier Science Ltd. 2017, Elsevier Science Ltd.

Furthermore, the same group investigated the influence of the BC on the water uptake of PA6 [81] by immersing samples in water at 85 °C for time intervals up to 28 h. The results were also compared with those obtained for talc-reinforced nylon composite at 20 wt.%. The addition of BC to PA6 caused a reduction of the water uptake during the conditioning process compared to the unfilled polymer. The impact strength remained almost unchanged even after conditioning, suggesting that the interaction established between BC and PA6 can restrict the chain mobility, thereby eliminating the effect of moisture on the polymer. The morphological analysis of the impact fracture surfaces, reported in Figure 7, showed relevant differences among the samples. In fact, in the conditioned PA6, the number of ridge-like structures and grooves with a lot of cracks increased with respect to the unconditioned sample, confirming the plasticizing effect exerted by water after conditioning. The PA6–talc samples after 28 days of conditioning exhibited the exposure of more talc particles on the surface. When the morphology of biochar–PA6 composites was observed, two distinct phases appeared, namely: Phase 1, where debonding of some of the BC particles from PA6 and pullout of the filler during impact fracture were visible, and Phase 2 that is similar to the unconditioned sample and in which the BC was wetted by the matrix and not clearly distinguishable. Finally, even if the performance of PA6–BC after conditioning was reduced, these composites presented better tensile and flexural strength values before and after conditioning, comparable moduli after conditioning, and lower density in comparison to PA6–talc samples, hence indicating the suitability of BC as a lightweight alternative to talc. 

In another work, the accelerated thermo-oxidative aging of PA6–BC was compared with other PA composites containing commercial talc and glass fiber used in the automotive industry [82]. The authors found that the glass-filled composites had the best mechanical performance, whereas both talc- and BC-containing composites displayed similar mechanical properties in terms of strength and ductility, with BC-filled composites being 11% less dense than talc-filled counterparts, the filler loading being equal. The mechanical performance (tensile and impact strength) of all the analyzed composites decreased after thermo-oxidative aging for 1000 h at 140 °C; however, the degradation of PA6–BC composites was more significant than that of the other composites. This finding was attributed to the physical structure of BC.

### 3.3. Polyester-Based Composites

Among polyesters, the literature reports a significant number of publications on the preparation and characterization of polylactic acid (PLA)–BC composites. Currently, PLA is utilized in many fields comprising biomedicine, textiles, films, decorative panels, electrical elements, and food packaging. However, due to its poor crystallization property, PLA presents low thermal stability, low toughness, and high brittleness, which restrict its extensive industrial application [83]. The use of BC as a filler can lead to growing market demand for PLA composites for higher potential applications such as transportation, automotive, electronics, and electromagnetic interference shielding [84].

In most of the existing research, the incorporation of BC into PLA has been considered as a method for enhancing the mechanical properties of this polymer [85,86,87,88,89,90,91,92,93,94,95]. In general, regardless of the nature of BC and its amount, an increase in the elastic modulus and a decrease in tensile strength, elongation at break, and impact strength were usually found [87,88,89,90,91]. The decrease in tensile strength could be attributed to stress transfer inefficiencies and the lack of homogeneous dispersion of BC particles in the matrix. 

Ho et al. [92] found that the maximum tensile strength, flexural strength, and ductility index of PLA–bamboo char composites prepared by extrusion molding were 43%, 99%, and 52%, respectively, higher than those of unfilled PLA but only when the content of bamboo char was below 7.5%; this finding was attributed to the homogeneous dispersion of BC within the matrix achieved below this loading. Qian et al. [93] prepared PLA composites reinforced with 1000 mesh ultrafine bamboo char. BC was efficiently dispersed in the PLA matrix, and the two phases had good interfacial interaction when BC content achieved 30 wt.%. Tensile strength and modulus increased as a function of the BC loading until 30 wt.% (for which 14 MPa and 558 MPa were measured, respectively), then slightly decreased values were registered. Similarly, for the same composite, the impact strength reached a maximum value of 20.50 J·m^−2^. It was shown that the elongation at the break of the composites was lower than that of unfilled PLA; however, the decrease in ductility became negligible for BC loadings beyond 10 wt.%. This tendency mainly resulted from the low aspect ratio of BC because of stress concentration during tensile deformation and breakage. 

Some efforts were also devoted to study the influence of interfacial bonding and BC surface area on the properties of PLA composites. In detail, some works reported the chemical modification of BC and/or PLA macromolecules with the hypothesis that the introduction of coupling agents, by enhancing the interfacial bonding between BC and polymer chains, improves the physico-mechanical and thermal properties of composites [87,88,89]. In general, it has been reported that the use of maleic anhydride functional groups grafted onto PLA (3 wt.%) had only a marginal impact on the mechanical properties of the resulting composites [88,89]. Salak et al. exploited a noncatalytic thermal esterification reaction of BC particles derived from kudzu after its thermal pyrolysis in the presence of phthalic anhydride, aiming at improving the compatibility of the filler with PLA [87,88,89]. The treatment caused further improvement in the physico-mechanical properties of PLA-BC composites. Furthermore, the pre-treatments were effective in enhancing the water or hydrolysis resistance of the composites. Quian et al. modified the BC surface with different concentrations of HNO_3_ and NaOH solutions. The HNO_3_ treatment induced the grafting of amino groups, while that with NaOH modified carbonyl and carboxyl groups on bamboo char. It was reported that the mechanical behavior depends on the concentration of the chemicals used for the treatment [95]. Finally, it was found that the functionalization of ultra-fine-bamboo char with (3-mercaptopropyl) trimethoxysilane favored its dispersion in PLA [94]. 

BC, in general, affected the thermal property of PLA by reducing its thermal stability (decrease of T_onset_ and T_max_) and its glass transition and melting temperatures. The detrimental effect of BC particles derived from different sources on the thermal and thermo-oxidative stability of PLA and other bio-polyesters matrices has already been reported in the literature and attributed to the catalytic effect of potassium, usually contained in BC, on the decomposition of the polymer matrix [85,90]. However, both hydrolytic and “back-bite” reaction mechanisms [96] may take place during the thermal degradation of PLA, owing to residual hydroxyl functionalities present on the BC surface [97]. Additionally, the introduction of BC was found to improve the mobility of PLA macromolecular chains, inducing a plasticization effect. 

Finally, abrasion resistance, wear resistance, and flammability of PLA–BC composites were thoroughly studied [98,99]. In particular, BC improved the tribological properties of the polymer matrix, decreasing the volume loss. This finding was attributed to the high stiffness and good dispersion of BC throughout the matrix [98]. Lastly, biochar was able to decrease the burning rate, thanks to its charring effect during combustion and to its barrier for the leading edge of the flame front [99].

BC was also used in combination with a third component for improving mechanical properties [94,100]. Sheng et al. [94] investigated PLA–bamboo char–cellulose nanowhisker composites. These ternary composites showed both mechanical strength and toughness, thanks to the presence of BC. Furthermore, BC and bamboo cellulose nanowhiskers exhibited synergistic effects, enhancing the toughness of the nanocomposites. A hypothesis for the observed reinforcing mechanism was provided, involving the formation of core-shell structures of ultra-fine BC particles and PLA during the tensile deformation of the composites, resulting in synergistic toughening and reinforcing effects. 

Biodegradable multiphase poly(lactic acid)-BC-graphite systems have been developed for applications in wearable/portable devices, radiation-sensitive electronics, and sensors and for producing EMI shielding materials [84]. Highly conductive composites (>30 S/m) with staggering shielding effectiveness (>30 dB) at very low film thickness (0.25 mm) were prepared. Furthermore, the potential of BC–PLA composites for fully biodegradable foaming applications was documented [101]. Nano- and micro-particles of BC derived from sludge, pistachio, and green waste were ground, sieved, ball-milled, and compounded with PLA at different ratios through an extrusion process. The PLA–BC extruded films were then foamed in a supercritical CO_2_ batch foaming process. The well-dispersed BC fillers at an appropriate concentration served as preferential nucleation sites with a lower energy barrier for nucleation, thus facilitating the cell nucleation process. In addition, the dispersed particles were able to simultaneously enhance the melt strength of the polymer matrix, thereby stabilizing nucleated cells by minimizing cell coalescence. 

Among polyesters, poly(butylene terephthalate) (PBT), poly(trimethylene terephthalate) (PTT) and poly(ethylene terephthalate) (PET) have been considered as suitable matrices for preparing composites with BC.

PBT composites, containing from 5 to 25 wt.% of commercial miscanthus grass pyrolyzed at 650 °C, were prepared by extrusion followed by injection molding [102]. Durability (aging at 155 °C for 1000 h) of the mechanical properties of these composites was evaluated and compared with that of PBT-based composites containing similar amounts of talc and glass fibers (GF). It was found that the tensile and flexural modulus of the PBT and its composites increased after thermo-oxidative aging. The PBT–BC composites showed the greatest decrease in mechanical strength compared to aged PBT–talc and PBT–GF counterparts. In general, the difference in maintaining the mechanical performance after aging for the PBT composites was mainly attributed to the difference in structure, aspect ratio, surface adhesion, and particle size of the used fillers. Thus, the best properties among the three investigated fillers were exhibited by GF due to high adhesion, strength, and stiffness. 

**Figure 8:**
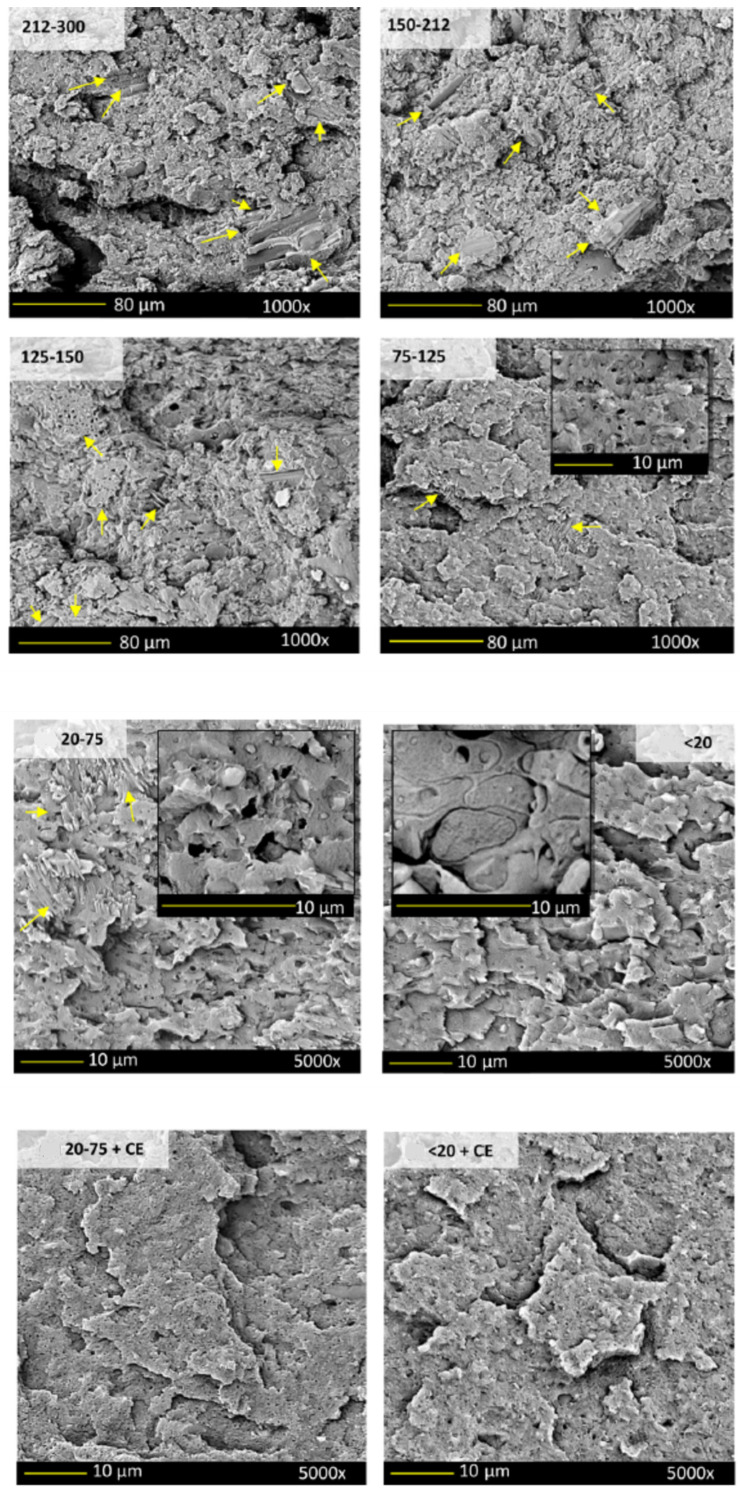
Morphology of composites with different size-fractionated BC (the arrows point to BC particles) [104]. Reproduced with permission from the American Chemical Society. 2016, American Chemical Society.

PPT [103] and PPT mixed with PLA and ethylene-methyl acrylate-glycidyl methacrylate (EMAGMA) [104] were also filled with BC. PTT [103] containing 20 wt.% of BC obtained by pyrolysis of lignin, with and without a chain extender additive, was extruded and injection molded. Reactive epoxy functional polymeric chains were used for improving the performance of the composites by maintaining the molecular weight and balancing the viscosity of PPT during processing. It was found that BC significantly improved the HDT and stiffness of the composite, whereas impact strength and yield elongation decreased. The chain extender improved the performance of the unfilled polymer but had no effect on the properties of PPT–BC composites. Nagarajan et al. [104] incorporated 15 wt.% of EMAGMA into PTT–PLA (30 wt.% of PLA) through melt compounding in an extruder, followed by injection molding. Commercial miscanthus-based BC produced through a low-temperature pyrolysis process was size-fractioned before use. Size ranges of 212−300, 150−212, 125−150, 75−125, 20−75, and <20 μm were obtained. In addition, an epoxy chain extender was employed. Different morphologies were observed as a function of the BC size and the presence of the chain extender that in turn influenced the mechanical performance of the composites. In detail, it was reported that the blend matrix retained its “primary sea-island” morphology with round domains of the PLA−EMAGMA dispersed in the composites when the size of the embedded BC was in the ranges of 75−125 and 20−75 μm (Figure 8). When the particle size was above 125 μm, BC acted as a barrier to the dispersion of the polymer components during processing. Using BC below 20 μm allowed achieving a good dispersion of the filler that probably stabilized the morphology, and the PLA-EMAGMA domains coalesced, as can be observed from the SEM pictures shown in Figure 8. Finally, the addition of an epoxy-based chain extender induced the suppression of coalescence and promoted the dispersion of the PLA−EMAGMA domains in much smaller and finer morphologies. The latter morphologies were favorable in improving the mechanical properties and in particular the impact strength with respect to the unfilled blend. Furthermore, the influence of the injection mold temperature (30, 60, and 90 °C) was evaluated, showing that a high temperature was responsible for the improvement in crystallinity that, in turn, influenced the HDT and increased the flexural strength and the stiffness of these composites.

Recently, the mechanical properties of virgin and recycled PET composites containing 10 and 20 wt.% of commercial miscanthus-based BC pyrolyzed at 650 °C were investigated [105]. The composites also contained 20 wt.% of a toughening agent (ethylene-butyl acrylate-glycidyl methacrylate, EBAGMA) and 1 phr of a chain extender (styrene-acrylic glycidyl methacrylate, SAGMA). The extrusion–injection molded samples were tested for their tensile and impact properties, and a full factorial design of experiment (DOE) was used for evaluating the influence of recycled PET content, BC loading, and chain extender presence on the final properties of the composites. It was found that the chain extender followed by the BC played the most dominant roles in influencing the Young’s modulus, tensile strength at yield, elongation at break, and impact strength. The optimal composite for attaining balanced properties was that containing up to 25 wt.% of recycled PET, 10 wt.% of BC, and the chain extender. Interestingly, the combination of the chain extender and the BC at 10 wt.% enhanced stiffness and tensile strength through a synergistic effect, providing a 278% increase of impact toughness with respect to the composite containing BC (without a chain extender).

### 3.4. Other Thermoplastic-Based Composites

The use of BC for modifying engineering amorphous polymers such as polycarbonates (PC) has not found a big interest in the current scientific research. Today in the literature, there are only two works by Andrzejewsk and co-workers [106,107], where a mixture of miscanthus-based biochar pyrolyzed at 900 °C (having a size of 400 µm) and polyacrylonitrile-based recycled carbon fibers (CF) were used as fillers. In the first work [106], to prevent or decrease the hydrolytic process of PC during the extrusion process, a chain extender was employed. The results indicated numerous drawbacks of the use of biochar filler alone, whereas the use of combined BC and CF improved the mechanical properties of the polymer matrix. In detail, in the composite containing both BC (10 wt.%) and CF (10 wt.%), the tensile modulus and strength increased by 35% and 270%, respectively, compared to the composite containing 20 wt.% of BC. A similar behavior was observed for the flexural properties (with increments of 16% for the modulus and of 305% for the strength). However, without the presence of a commercial epoxy chain-extender additive, the biochar intensified the phenomenon of hydrolytic degradation, which further worsened the thermo-mechanical stability of the composites.

To limit the degradation of the PC during blending, the same group exploited the addition of acrylonitrile butadiene styrene (ABS) to the PC for preparing composites [107]. First, composites based only on PC and ABS were prepared and characterized. The materials were reinforced with different fillers, namely biochar, carbon fiber, and biochar–carbon fiber mixtures. When fillers were used alone, their loading was set at 10 and 20 wt.%. Then, biochar and CFs were used together (at 10 and 20 wt.%), and the results on the mechanical properties were compared with those of the single-filled systems. The results confirmed a worsening of the rheological properties, a reduction of viscosity, and a decrease in the glass transition temperature and in the mechanical properties for the composites due to processing. However, the addition of ABS to the PC matrix impeded the hydrolytic degradation of the PC–BC composites. From all the investigated mechanical properties and in particular from the impact resistance values, it was clear that the concurrent presence of biochar and CFs provided beneficial effects to the prepared blends. Finally, the results of the coefficient of linear thermal expansion (CLTE) measured in different directions showed a significant improvement in structure uniformity for the composites containing biochar and CFs, indicating a minimal risk of stress concentration for these materials.

In a further research effort, Nan et al. [108] used polyvinyl alcohol (PVA) as a model polymer matrix for studying the electrical conductivity and mechanical properties of biochar. In their first work, the authors prepared wood biochar–PVA composite films and investigated their electrical, mechanical, and thermal properties. Composite films were obtained by casting, adding 2, 6, and 10 wt.% of commercial biochar derived from wood to a 10 wt.% PLA solution. The solution was sonicated for improving the dispersion. The obtained results indicated that the conductivity of PVA–biochar composite films increased with increasing the biochar loading and reached the value of 1.8 nS/m for 10 wt.% biochar loading. The introduction of biochar increased the elastic modulus and thermal stability but reduced the tensile strength, storage modulus, T_g,_ and T_m_.

Furthermore, Nan et al. [109] investigated the behavior of these PVA composite films as pressure sensors; the BC loading was 8, 10, and 12 wt.%. They found that the increase in biochar content from 8 to 12 wt.% significantly improved the conductivity and piezoresistive effect of the PVA–biochar sensors. Then, by increasing the pressure from 0 to 358 kPa, the resistance of the composite sensors gradually decreased, with a reduction of 99% for the composite loaded with 12 wt.% of BC. This decrease indicated that the applied pressure induced the film deformation, and this feature was responsible for the formation of a higher number of conductive paths, which increase the conductivity. Similar results were achieved by Bartoli et al. [110], who dispersed biochar derived from waste cotton into PVA, achieving a conductivity of up to 16 S/m under a pressure of 750 bar.

Starch was mixed with biochar mainly with the purpose of biomass densification for obtaining pellets; only Hu et al. [111] studied the nano-mechanical properties of these composites by atomic force microscopy (AFM). The work mainly aimed at demonstrating the feasibility of AFM for visualizing the Young’s modulus of the composite. Composites containing rice husk biochar at different loadings (namely 0.5, 1, 2, and 5 wt.%) were obtained by casting. Components were dispersed in water and mixed with glycerol and acetic acid at high temperature for gelatinization. It was found that the Young’s modulus of the composites decreased with increasing the BC loading because of weak bonding among the components. In another work [112], thermoplastic starch was used with polycaprolactone (PCL, blended with 50 wt.% of starch) for incorporating biochar obtained from waste coffee grounds (selected loading: 10, 20, and 30 wt.%). It was found that by adding biochar at 10 wt.%, the elastic modulus increased, and the tensile strength was slightly reduced. By increasing the biochar content, there was no significant effect on the tensile strength and elastic modulus of the material. However, the elongation at break was drastically reduced with the incorporation of biochar. Interestingly, it was manufactured as a male mold of a coffee cup lid, demonstrating the potential application of this biodegradable composite material.

Among polyhydroxyalkanoates, only poly(3-hydroxybutyrate-co-3-hydroxyvalerate) (PHBV) was used as a matrix for biochar composites production [113]. The authors prepared composites of PHBV filled with miscanthus biochar (PHBV/MB) at 10, 20, and 30 wt.% loadings with the aim of improving tensile, flexural, and impact properties. In addition, considering that the PHBV has a high production cost, the use of biochar could reduce the amount of polymer for preparing cheaper composites. 

The authors found that the elastic and flexural modulus increased, though the tensile and flexural strength and elongation at break decreased at any biochar loading. The biochar slightly decreased the thermal stability of the PHBV. On the other hand, the HDT increased, and the coefficient of linear thermal expansion decreased with increasing the biochar loading, suggesting an enhanced dimensional stability of the material due to the addition of the rigid biochar particles.

### 3.5. BC Composites: Thermosetting matrices

#### 3.5.1. Epoxy Resin-Based Composites

**Figure 9:**
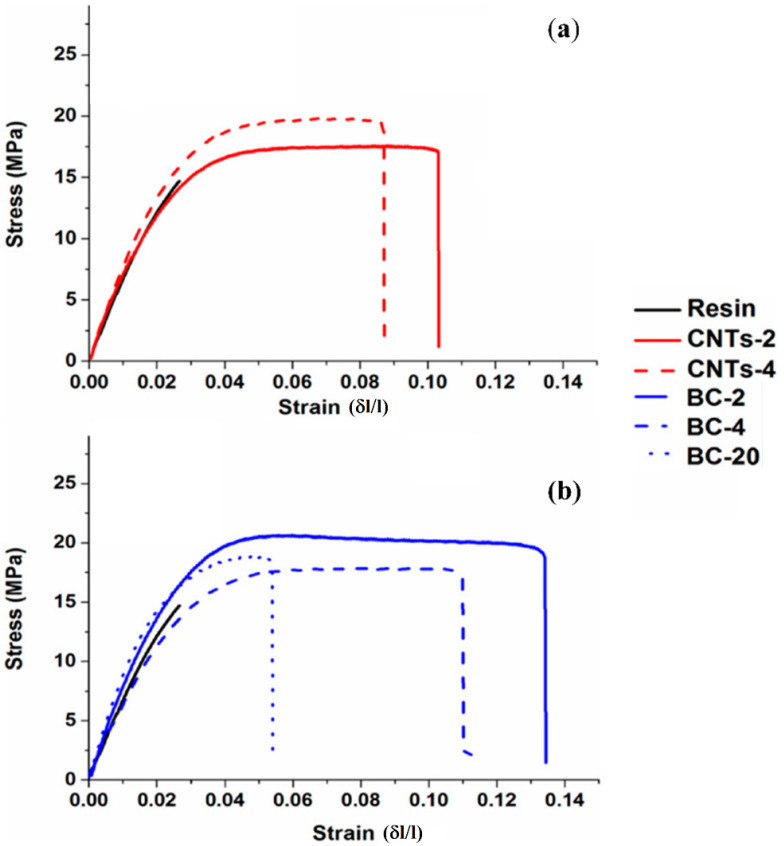
Stress–strain behavior of neat epoxy resin (black curve) compared to composites containing: (**a**) multiwalled carbon nanotubes at 2 wt.% and 4 wt.%; and (**b**) BC at 2 wt.%, 4 wt.% and 20 wt.% [117]. Reprinted under CC BY 4.0 license.

Among different thermosetting hosts, epoxy resins are one of the most studied due to the wide range of applications in many strategic industrial productions ranging from the aeronautic to the automotive sectors [114]. These applications are generally based on carbon, glass fibers, and CNTs reinforced materials [115,116]. The use of BC as filler for epoxy resin should face the performance achievable with such high-tech fillers. A first comparative and comprehensive study was reported by Khan et al. [117], who investigated the mechanical performance through tensile tests of epoxy composites containing different amounts of CNTs and maple-derived BC (Figure 9).

A BC concentration not exceeding 2 wt.% improved the overall mechanical behavior of epoxy-based composites, showing better results compared to the incorporation of CNTs. Nonetheless, a higher filler content was required to reach the electrical conductivity achieved by using CNTs. A 20 wt.% of BC produced at 900 °C displayed a conductivity in AC mode higher than that observed for the composites containing 4 wt.% of CNTs. Similarly, Giorcelli et al. [118] filled an epoxy matrix with 15 wt.% of coffee-derived BC, measuring a conductivity of 36 S/m. This value was much higher than that achieved by using the same amount of carbon black; however, the high filler loading decreased the elongation at break by 47% and doubled the Young’s modulus. The increment of brittleness was due to the limited mobility of the matrix that was unable to efficiently redistribute the applied stresses. This was also reported in another work [119], where a simple model to describe the properties of composites by using the mixing rule was developed. The authors clearly demonstrated that 2 wt.% of BC was the best filler concentration for the optimization of the mechanical behavior of the composites.

**Figure 10:**
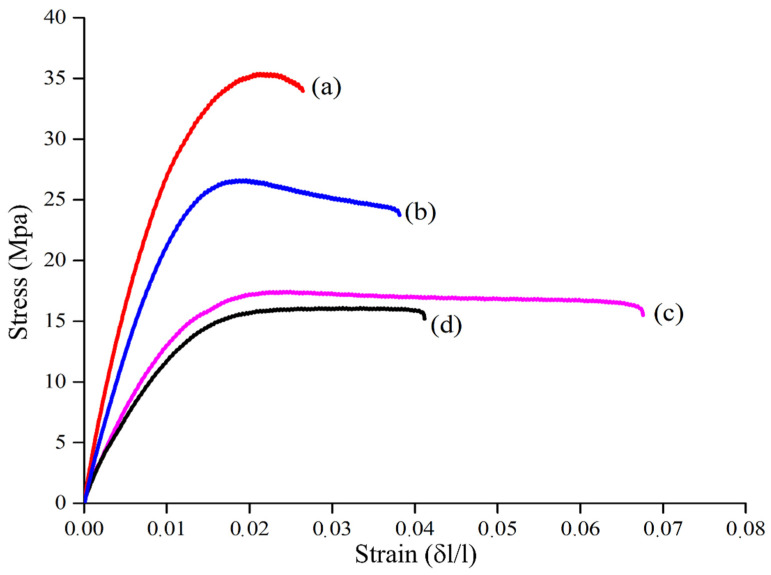
Stress–strain curves of composites filled with 2 wt.% of BC derived by: (a) wheat; (c) *Mischantus*; stress–strain curve of composites filled with 2 wt.% of carbon black Vulcan ^®^ 9 N115 (b); and of unfilled resin (d). Reprinted from [120] under CC BY 4.0 license.

Concentration was not the only parameter that deeply affected the outputs of epoxy resin-BC systems. By using a filler concentration of 2 wt.%, Bartoli et al. [120] explored the effect of feedstock used for the BC production on the stress–strain curves (Figure 10).

As observed through a rough comparison, the BC-containing composites were able to induce greater increments in stiffness (Curve (a) in Figure 10) and in elongation (Curve (c) in Figure 10), compared to carbon black (Curve (b) in Figure 10). Furthermore, a surprisingly different behavior from brittle to ductile was observed for the different biochars. The authors suggested that mechanical outputs of composites were due to the simultaneous effect of both size distribution and surface roughness of the particles. Accordingly, they reported that small and rougher BC particles promoted an increment of stiffness, while big particles with smooth surfaces induced an increment of elongation at break.

**Figure 11:**
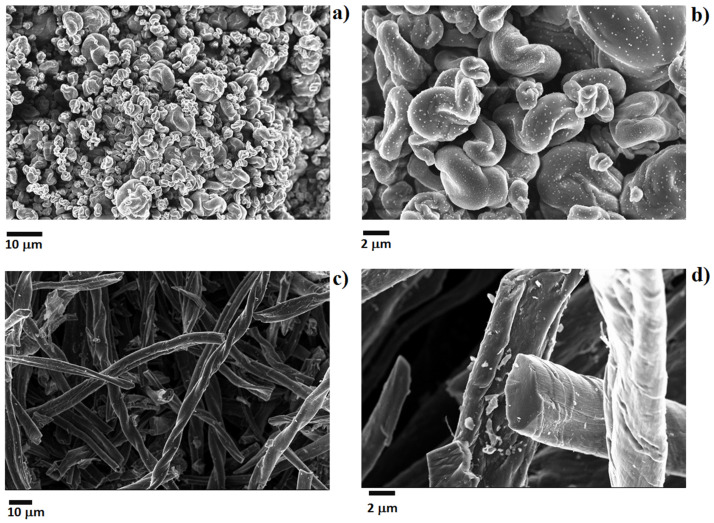
Field emission scanning microscope captures of BC particles produced by pyrolysis at 400 °C of (**a**,**b**) waste cotton fibers and (**c**,**d**) cellulose nanocrystals [121]. Reprinted with permission from Wiley Periodicals, LLC.

More general studies about the influence of BC particles shape were reported by Bartoli et al. [121,122] by using cellulose-derived micrometric carbon rods and spheres, as shown in Figure 11.

By using these materials, the authors were able to evaluate the effect of shape excluding the influence of the porosity of wood-derived BC. In particular, the best mechanical properties were obtained by using up to 2 wt.% of BC loading. Remarkably, the elongation at break decreased by increasing the filler loading only when carbonaceous rods were employed. This finding was attributed to a better dispersibility of spheres and to a network formed by rods that reduced the macromolecular chain mobility. 

The performance of BC-containing epoxy composites are also related to the thermals stage and ramps adopted during BC production. As reported by Bartoli et al. [123], the highest temperature reached during carbonization of olive trunks was a crucial parameter for tuning the mechanical features of the resulting composites. The authors studied epoxy composites containing 2 wt.% of BC produced at four different temperatures (namely, 400 °C, 600 °C, 800 °C, and 1000 °C), and using three heating rates (5 °C/min, 10 °C/min, and 50 °C/min). The obtained data showed an increase of Young’s modulus of the composites by about 35% when BC was produced at 400 °C or 600 °C and a heating rate of 50 °C/min was employed. Conversely, the incorporation of BC produced at 1000 °C slightly decreased the stiffness, while the elongation at break improved by about 50%. Furthermore, BC produced using temperatures from 400 °C to 800 °C improved the ultimate tensile strength by about 30%. It was suggested that higher temperatures promote the formation of greater aromatic domains on BC particles that interact with the aromatic moieties of resin through weak interactions, while the tailored surface of BC produced at lower temperatures is able to create hydrogen bonds with the matrix. The greater interaction promoted by low temperature pyrolysis improved the particle–matrix adhesion with a less efficient ability of the polymer host to redistribute the stresses due to a reduction of polymer chains mobility.

As reported by Oral [124], the incorporation of up to 30 wt.% of BC into an epoxy resin remarkably increased the elastic modulus (by about 90%). The authors evaluated the elastic moduli by using for the first time the elastic constants ultrasonic pulse echo overlap method, suggesting, without further insight, that data collected could be correlated to interatomic forces.

Another interesting application of BC is represented by its use as a toughening agent for fibrous-based epoxy composites. Although this application is very promising, the literature lacks studies specifically devoted to this topic. The first research ever reported was about BC added to a glass fiber-reinforced epoxy matrix by Dahal et al. [125]. The authors investigated the effect of BC produced by conversion of spruce wood pellets pyrolyzed at 450 °C on E-glass fibers epoxy composites. They reported a relevant increase of storage modulus by about 4000 MPa when 10 wt.% of BC was incorporated into the epoxy matrix. This behavior was ascribed to the modifications of interfacial properties between glass fibers and polymer with an improvement of grip promoted by the carbonaceous filler. 

Similarly, Matykiewicz [126] added BC to an epoxy matrix employed for producing carbon fiber-based composites. The authors demonstrated a significant increase of the storage modulus (by around 39%) through the addition of 10 wt.% of BC; meanwhile, the elongation at break did not remarkably change with respect to BC loading. An 18% increase of flexural strength and an 11% decrease of flexural modulus were observed. These findings were attributed to the rigid BC with a high surface that improved the interlocking of resin and fibers.

Zuccarello et al. [127] reported an increase of ultimate tensile stress and strain by about 55% and 250%, respectively, by adding 2 wt.% BC obtained from Mischantus pyrolyzed at 550 °C to agave fibers epoxy composites. Nonetheless, the greatest improvements were achieved considering the specific failure energy fatigue life performance that became 5 times and 3 orders of magnitude bigger with respect to the composites without BC. These findings were ascribed to BC that improved the grip between the fibrous filler and the polymeric host.

#### 3.5.2. Unsaturated Polyester Resin-Based Composites

Another relevant class of thermosetting matrices is represented by unsaturated polyester resins [128] that are largely used as construction materials [129,130].

Akaluzia et al. [131] investigated the effect of the incorporation of hardwood BC (loadings ranging from 5 to 30 wt.%) on hardness and impact strength of an unsaturated polyester resin. The obtained results showed an increment of impact energy by about 15%, while hardness increased by 100%.

A more detailed study was reported by Sundarakannan et al. [132] by using cashew nutshell BC produced at 500 °C as filler for an unsaturated polyester resin, at different loadings (from 5 up to 15 wt.%). The authors reported an appreciable increase of hardness, tensile, and impact strength compared with the unfilled matrix, by 37, 21, and 41%, respectively. The authors suggested that the brittle fracture of the materials was due to the poor interactions between BC and the polymer network. Noticeably, the maximum flexural strength was reached by using a BC loading of 15 wt.%. The improvement of up to 40% was due to the high uniform distribution amount of BC particles that promoted the resistance against bending solicitation.

A tribological study on BC-based unsaturated polyester resins was reported by Rajadurai et al. [133], who tried to correlate the tribological behavior with BC particle size distribution. The authors showed that the specific wear rate and friction coefficient of the composite decreased with increasing the BC loading and decreasing its particle size. The best result was achieved by using 2.5 wt.% of BC with an average size of 45 nm, which decreased the friction coefficient and wear rate by 56 and 46%, respectively. The lubricant effect of BC was the same observed by using other carbonaceous fillers such as nanographite [134] or graphene [135].

Similar results were achieved by Richard et al. [136] by using a finely milled red mug-derived BC in an unsaturated polyester resin. 

#### 3.5.3. BC-Rubber Composites

BC has also been used after mixing with nanosilica as filler for poly(styrene)-poly(butadiene) rubber by Peterson et al. [137]. The authors tried to replace carbon black with BC derived from maple pyrolysis in a formulation very close to that used to produce tires [138]. As reported, BC particles with an average size of over 10 μm imparted properties far lower than a commercial carbon black, while a size reduction of the filler promoted a general improvement of the mechanical features of composites. The authors claimed an increase of elongation at break and of toughness by 31 and 24%, respectively, without any loss of tensile strength compared with carbon-black-based composites. This was most probably due to a good dispersion and the sub-micrometric size of the employed BC.

Silicon rubber was also selected as a polymer matrix for the production of BC-based composites as reported by Giorcelli et al. [138]. The authors used an olive-derived BC thermally annealed at 1500 °C to produce a highly conductive filler able to undergo a fully reversible elastic deformation by applying a pressure strain up to 40 MPa.

## 4. A Comparative Perspective on BC and Other Carbonaceous Fillers

Carbonaceous fillers comprise a very wide range of materials from nanostructured species (i.e., CNTs, graphene) to carbon fibers with unique features and both advantages and disadvantages, as summarized in Table 1. In this vast realm, BC represents an outsider, freshly appearing on the scene. BC has generally found applications in environmental sciences and as electrode materials due to its surface and bulk features (i.e., conductivity, surface area, residual groups) [139]. The use of BC as a filler for polymer composites for different applications (automotive, electrical, and engineering purposes, among others) is a relatively new field opened up with by the outcomes of the Bhattacharyya research group only in 2015 [42]. Since then, BC has faced competition with high-performing fillers that can induce strong enhancements of either mechanical or electrical properties even at low concentrations [140,141] in all the fields that are traditionally occupied by carbonaceous materials. Nevertheless, materials such as CNTs and graphene are very expensive, and their dispersion into polymeric matrices is quite often difficult [142]. An efficient dispersibility for these fillers could be achieved only through advanced modifications that lead to further cost increments [143,144]. Compared to these carbonaceous nanofillers, BC is far less expensive and can easily be dispersed in almost all the polymeric matrices. Generally, though BC requires higher loadings to reach comparable performance with CNTs and graphene, its very low cost [145] makes it a reliable solution. In fact, despite the expected revolution, graphene and graphene oxide do not develop from research (i.e., lab-scale) and small productions. In 2009, Segal et al. [146] stated that the world was ready for the ton-scale production of graphene, but in 2020, single layer graphene was still sold at USD 230/cm^2,^ and graphene oxide costs USD 140/kg [147]. Pricing for research grade nanotubes ranges from around USD 5/g for MWCNT to USD 75/g for SWCNT [148]. Carbon fiber cost is around USD 18/kg. Carbon black (CB) is with a price four times higher than that of BC. (the CB price is about USD 1.2/kg and Biochar is sold at USD 0.35/kg) [149].

Considering the abovementioned issues, BC appears to be a rising star in the materials science field, but it is still struggling to move from lab-scale to industrial applications. This is mainly due to the competition with the real ruler of carbon-based composites, i.e., carbon black. Carbon black has a solid industrial reputation for plenty of commodities such as tires and ink production [150]. Nonetheless, BC will eventually win the competition with carbon black thanks to its greater sustainability and reduced environmental impact [151,152]. 

BC is able to provide the composites with sustainability combined with tailored features. These characteristics will lead biochar to major breakthroughs in the market because of the increased attention to environmental issues of all major stakeholders [153].

## 5. Conclusions and Future Perspectives

The exploitation of BC as a multi-functional filler in polymer-based composites has attracted great attention from the scientific community in recent years due to its excellent potential in developing sustainable and high-performance materials. The main benefits in using BC as a filler for the production of polymer-based composites are related to the possibility of achieving improved mechanical properties, electrical conductivity and thermal stability through the introduction of a sustainable and renewable material derived from valorization processes of post-consumer wastes and is fully included in the new circular economy strategies. 

Studies concerning the evaluation of the final performance of BC-polymer composites have demonstrated the effectiveness of BC derived from different sources for being used as reinforcing filler for a wide variety of either thermoplastics or thermosets. 

From a general point of view, materials with improved tensile and flexural properties, enhanced impact strength, and increased HDT can be achieved upon the introduction of BC particles obtained through proper production processes. Furthermore, BC was found to be able to impart electrical conductivity and flame retardance to either thermoplastics- or thermosets-based systems, revealing its potential in substituting traditional fossil-fuel derived and expensive carbonaceous fillers. In fact, compared to CNTs and graphene, BC is far less expensive and can be easily dispersed in almost all polymeric matrices. However, though BC-containing composites with very low particle loadings (a few wt.%) showed mechanical properties similar or even superior to those of analogous CNT- and/or graphene-filled systems, higher contents are required to reach comparable electrical performance. Interestingly, the strategy of utilization of BC in polymer composites allows supporting the global sustainable development and the circular economy approach through the valorization of post-production and post-industrial residue wastes. Nevertheless, BC/polymer composites are still at the development stage, and a great research effort is required to gain further insights into the production structure properties relationships of BC particles; in fact, the huge variability of the starting biomass sources, as well as the different exploitable operative conditions during the production process, bring about to the obtainment of BCs with heterogeneous characteristics, possibly affecting the uniformity of the performance of the resulting composites. In addition, the high moisture absorption of BC limits several of its applications. For these reasons, more research is needed to gain a complete understanding of the long-term performance of BC-containing composites at the molecular level. For example, their long-term viability (i.e., weathering and UV resistance) as well as the mechanism of degradation remain unknown.

Some possible future work perspectives in the field could include the reduction of BC size from macro-/micro- to nano to better understand its potential as a filler and the improved interaction of BC with polymer matrices with the addition of compatibilizers or by chemical modification (which can increase the mechanical properties of the composite afterward).

Finally, the main challenges surely include: (i) improving the process output sustainability and economic feasibility so that scale production of BC in bio-composite applications may be carried out in the future; and (ii) using BC to completely replace present crude oil-based CB reinforced rubber materials. Indeed, the difficulty in managing the size and shape of BC produced from different wastes and biomasses requires the development of a more innovative pyrolysis processes comprising suitable treatments and procedures. 

## Figures and Tables

**Table 1:** A brief overview of the main advantages and disadvantages related to composite production using carbonaceous fillers.

Filler	Advantages	Disadvantages
CNTs	Increment of mechanical properties with low filler loading Low percolation threshold	High cost Complex production and purification Poor dispersibility into polymers
Graphene	Increment of mechanical properties with low filler loading Low percolation threshold Production of transparent composites	Very high cost Complex production and purification Poor dispersibility into polymers
Graphene oxide	Increment of mechanical properties with low filler loading Production of transparent composites	High cost Poor electrical properties
Carbon fibers	Superior mechanical and electrical performance Resistance to harsh environment	Very high price Complex production Delamination phenomena Difficult to dispose
Carbon black	Very cheap Great annual-based production Tunable properties of related composites Highly compatibility with several polymers	Oil derived
Biochar	Cheap Tunable properties of related composites Biomass and waste derived Highly compatibility with several polymers	Poor annual-based production Poorly standardized production

## Data Availability

Not applicable.
